# Choosing Wisely in intensive care medicine

**DOI:** 10.5935/0103-507X.20200003

**Published:** 2020

**Authors:** Suzana Margareth Lobo, Ciro Leite Mendes, Ederlon Rezende

**Affiliations:** 1 Hospital de Base, Faculdade de Medicina de São José do Rio Preto - São José do Rio Preto (SP), Brazil.; 2 Hospital Universitário, Universidade Federal da Paraíba - Campus I - João Pessoa (PB), Brazil.; 3 Hospital do Servidor Público Estadual “Francisco Morato de Oliveira” - São Paulo (SP), Brazil.

Intensive care medicine is a specialty in which the results are closely related to the ability to organize and work as a team. While technological advancements offer a wide variety of tests and a myriad of possible treatments and procedures for critically ill patients, this large arsenal of options is often misused, which in addition to not providing significant benefits to patients can cause unnecessary risk and harm. Based on these principles, the American Board of Internal Medicine Foundation (ABIM) began a campaign in 2012 to identify clinical practices that should be questioned to make physicians aware of the importance of using only interventions and procedures that are indicated for the patient and that do not put him or her at risk. For this purpose, a list of items considered most relevant to conscious decision-making was developed and was named *Choosing Wisely*.^(1)^ Since then, numerous medical societies around the world have started to make their lists to prompt discussions of and providing warnings about the main practices to be questioned and the importance of always using the utmost discernment. The following are considered basic principles of the campaign: it must be led by physicians, the choices should be patient-centered, there must be multi-professional participation, the campaign should be evidence-based, and the selection process must be transparent.^(2^

The *Associação de Medicina Intensiva Brasileira* (AMIB) appointed a group of experts to prepare the recommendations. Initially, the experts elaborated 28 recommendations and, by using the modified Delphi method, those that reached more than 80% consensus among them were excluded. In the end, there were ten recommendations, which the members voted on electronically through the association’s website (https://amorintensopelavida.com.br/choosing/). Members received an invitation to vote by email and were identified by their Natural Person Registration (CPF - *Cadastro de Pessoa Física*) number at the time of voting to avoid duplication. A total of 1,754 members from all regions of the country participated, which represented approximately 30% of AMIB members. The five recommendations that received the most votes were chosen by more than 50% of the participants, as shown in figure 1.

**Figure 1:**
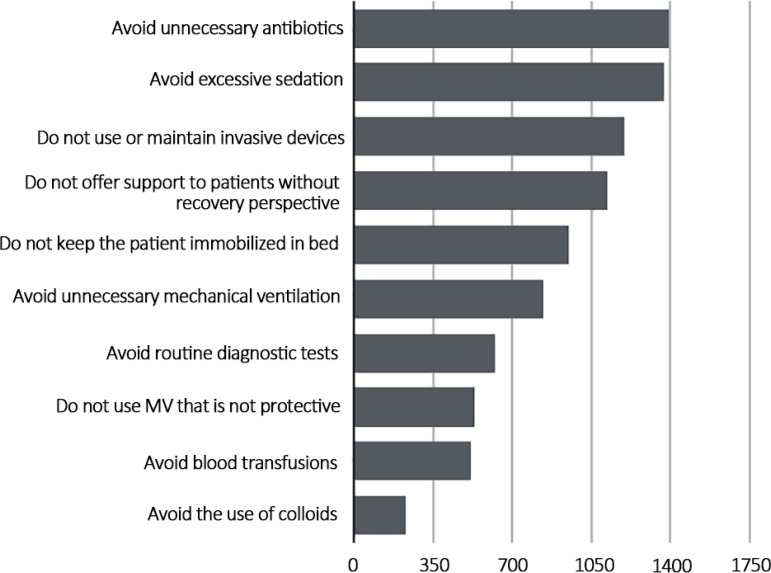
List submitted to the voting of members of the *Associação de Medicina Intensiva Brasileira* and votes received. MV - mechanical ventilation.

## TOP RECOMMENDATIONS

### 1 - Do not use or maintain unnecessary antibiotics

The use of antibiotics should be restricted to patients with infection, always follow the clinical criteria, be used for the shortest time possible, and according to the best evidence. Broad-spectrum antimicrobial agents should be deescalated or discontinued once cultures are available. Approximately 10 years ago, Boucher et al.^(3)^ called attention to the impact of the use of unnecessary antibiotics on the emergence of multiresistant bacteria, which became known as ESKAPE, an acronym for *Enterococcus faecium, Staphylococus aureus, Klebsiella pneumoniae, Acinetobacter baumanii, Pseudomonas aeruginosa* and *Enterobacter* species. Studies such as EPIC II (Extended Prevalence of Infection in Intensive Care) have shown that the number of patients admitted with an infection diagnosis to intensive care units (ICUs) in Latin America is higher than that in other regions of the world and that these patients have higher mortality.^(4)^

### 2 - Do not use excessive sedation

Limiting the use of sedation to patients with appropriate indications, using as few sedatives as necessary to maintain patient comfort, and using scales to systematically evaluate the titration of the drugs being used show improved clinical outcomes.^(5)^ Among the numerous pieces of evidence, we highlight the ABC trial, which combined daily sedation interruption with the performance of spontaneous breathing tests and compared this strategy to the standard treatment strategy; the former showed improvement on all outcomes, including more time free from mechanical ventilation, shorter stays in the ICU and in the hospital, and above all, lower mortality at a 1-year follow-up.^(6)^

### 3 - Do not keep patients immobilized in bed without a clear indication

The immobilization of critically ill patients is associated with a higher incidence of complications and longer hospital stays. There is evidence that early mobilization accelerates recovery from critical illness and improves the quality of life during hospitalization and after discharge. Accordingly, the study by Schweickert showed that daily discontinuation of sedation combined with physical and occupational therapy had an important impact on the recovery and functional capacity of patients at the time of hospital discharge.^(7)^

### 4 - Do not use or maintain invasive devices unnecessarily

The insertion and maintenance of invasive devices should always occur in a restrictive manner according to precise criteria. Routine surveillance is indicated to avoid as much as possible the insertion and prolonged use of tracheal and enteral tubes, as well as catheters and drains. There is evidence that the use of invasive devices is associated with infections and that their duration of use is prolonged either for the convenience of the professional team or because of a lack of protocols for their removal.^(8,9)^ Pronovost and colleagues conducted a study in ICUs in the state of Michigan, in which they evaluated daily whether any catheters or tubes that were no longer necessary could be removed showing a significant decrease in bloodstream infections.^(10)^

### 5 - Do not offer Advanced Life Support in the end-of-life for patients that are likely to die

The establishment or maintenance of advanced support for critically ill patients with a high probability of death or significant sequela should be avoided unless the possibility of establishing palliative care is considered. Clinical decisions within this context should always be made while respecting the expressed will of patients or their family members after extensive dialogue and consensus. In a recent study on terminality in the ICU by the World Federation of Societies of Intensive and Critical Care Medicine demonstrated wide variability in end-of-life practices in ICUs and the need of systematization.(^(10^

Quality improvement, cost reduction, value maximization and better care for chronic critically ill patients are current challenges of our specialty. To this end, wiser choices can certainly contribute in a significant manner. Publicize the list, make it part of your daily routine, include it in the checklists used in your unit, and discuss these choices during multi-professional visits. Doing so we will advance a national dialogue on avoiding unnecessary treatments and procedures I order to achieve better outcomes as a result.

## THE GROUP THAT ORGANIZED THE CHOOSING WISELY RECOMMENDATIONS OF AMIB:

Ederlon Alves de Carvalho Rezende, Mirella Cristine de Oliveira, Cristiano Augusto Franke, Marcos Knibel, Nelson Akamine, Álvaro Rea-Neto.

## References

[1] Cassel CK, Guest JA (2012). Choosing wisely: helping physicians and patients make smart decisions about their care. JAMA.

[2] American Board of Internal Medicine Foundation Choosing Wisely.

[3] Boucher HW, Talbot GH, Bradley JS, Edwards JE, Gilbert D, Rice LB (2009). Bad bugs, no drugs: no ESKAPE! An update from the Infectious Diseases Society of America. Clin Infect Dis.

[4] Vincent JL, Rello J, Marshall J, Silva E, Anzueto A, Martin CD, Moreno R, Lipman J, Gomersall C, Sakr Y, Reinhart K, EPIC II Group of Investigators (2009). International study of the prevalence and outcomes of infection in intensive care units. JAMA.

[5] Kress JP, Pohlman AS, O'Connor MF, Hall JB (2000). Daily interruption of sedative infusions in critically ill patients undergoing mechanical ventilation. N Engl J Med.

[6] Girard TD, Kress JP, Fuchs BD, Thomason JW, Schweickert WD, Pun BT (2008). Efficacy and safety of a paired sedation and ventilator weaning protocol for mechanically ventilated patients in intensive care (Awakening and Breathing Controlled trial): a randomised controlled trial. Lancet.

[7] Schweickert WD, Kress JP (2011). Implementing early mobilization interventions in mechanically ventilated patients in the ICU. Chest.

[8] Ziegler MJ, Pellegrini DC, Safdar N (2015). Attributable mortality of central line associated bloodstream infection: systematic review and meta-analysis. Infection.

[9] Klompas M, Anderson D, Trick W, Babcock H, Kerlin MP, Li L, Sinkowitz-Cochran R, Ely EW, Jernigan J, Magill S, Lyles R, O'Neil C, Kitch BT, Arrington E, Balas MC, Kleinman K, Bruce C, Lankiewicz J, Murphy MV, E Cox C, Lautenbach E, Sexton D, Fraser V, Weinstein RA, Platt R, CDC Prevention Epicenters (2015). The preventability of ventilator-associated events. The CDC Prevention Epicenters Wake Up and Breathe Collaborative. Am J Respir Crit Care Med.

[10] Pronovost P, Needham D, Berenholtz S, Sinopoli D, Chu H, Cosgrove S (2006). An intervention to decrease catheter-related bloodstream infections in the ICU. N Engl J Med.

[11] Myburgh J, Abillama F, Chiumello D, Dobb G, Jacobe S, Kleinpell R, Koh Y, Martin C, Michalsen A, Pelosi P, Torra LB, Vincent JL, Yeager S, Zimmerman J, Council of the World Federation of Societies of Intensive and Critical Care Medicine (2016). End-of-life care in the intensive care unit: Report from the Task Force of World Federation of Societies of Intensive and Critical Care Medicine. J Crit Care.

